# Nuclear quantum memory for hard x-ray photon wave packets

**DOI:** 10.1126/sciadv.adn9825

**Published:** 2024-06-26

**Authors:** Sven Velten, Lars Bocklage, Xiwen Zhang, Kai Schlage, Anjali Panchwanee, Sakshath Sadashivaiah, Ilya Sergeev, Olaf Leupold, Aleksandr I. Chumakov, Olga Kocharovskaya, Ralf Röhlsberger

**Affiliations:** ^1^ Deutsches Elektronen-Synchrotron DESY, Notkestr. 85, 22607 Hamburg, Germany.; ^2^ The Hamburg Centre for Ultrafast Imaging CUI, 22761 Hamburg, Germany.; ^3^ Department of Physics and Astronomy and Institute for Quantum Science and Engineering, Texas A&M University, College Station, TX 77843, USA.; ^4^ Helmholtz-Institut Jena, Fraunhoferstr. 8, 07743 Jena, Germany.; ^5^ GSI Helmholtzzentrum für Schwerionenforschung GmbH, Planckstr. 1, 64291 Darmstadt, Germany.; ^6^ ESRF - The European Synchrotron, CS40220, 38043 Grenoble Cedex 9, France.; ^7^ Friedrich-Schiller Universität Jena, Institut für Optik und Quantenelektronik, Max-Wien-Platz 1, 07743 Jena, Germany.

## Abstract

Optical quantum memories are key elements in modern quantum technologies to reliably store and retrieve quantum information. At present, they are conceptually limited to the optical wavelength regime. Recent advancements in x-ray quantum optics render an extension of optical quantum memory protocols to ultrashort wavelengths possible, thereby establishing quantum photonics at x-ray energies. Here, we introduce an x-ray quantum memory protocol that utilizes mechanically driven nuclear resonant ^57^Fe absorbers to form a comb structure in the nuclear absorption spectrum by using the Doppler effect. This room-temperature nuclear frequency comb enables us to control the waveform of x-ray photon wave packets to a high level of accuracy and fidelity using solely mechanical motions. This tunable, robust, and highly flexible system offers a versatile platform for a compact solid-state quantum memory at room temperature for hard x-rays.

## Teaser

Doppler-shifted nuclear resonant absorbers form a nuclear frequency comb, enabling a quantum memory in the hard x-ray range.

## INTRODUCTION

Photon wave packets are fast and robust carriers of quantum information encoded in various degrees of freedom, including photon number, polarization, and waveform. However, their fast propagating nature renders storage of quantum information challenging—an instrumental part not only for synchronization processes in quantum communication networks but also for the realization of quantum repeaters (*1*–*5*). Thus, there is a quest for quantum memories that store the photon wave packet or its specific quantum information and retrieve them without distortion at a specific time (*1*, *5*, *6*). In the visible wavelength regime, several protocols have been developed, which map the fast photonic wave packets to coherent collective quantum excitations in “slower” matter systems, such as ensembles of atoms, molecules, rare-earth ions in crystals, or color centers in diamond (*1*, *4*–*9*). A controlled de-excitation of the system results then in the release of the wave packet.

The step to shorter wavelength regimes, however, has not been achieved thus far, despite its benefits. Using x-rays would allow us to develop more compact and flexible quantum memory protocols due to x-rays being potentially focusable down to the ångström scale, about four orders of magnitude shorter than wavelengths of visible photons, and demonstrating higher penetration depths in many materials. Moreover, at those high energies, efficient low-noise single-photon detectors are available (*10*). To date, hard x-ray quantum memories have only been discussed theoretically (*11*, *12*). Mössbauer nuclei with their energetically sharp resonances are a potential material system. The prominent 14.4 keV nuclear resonance of ^57^Fe was already used to establish many quantum optical phenomena in the x-ray regime [see (*13*) and references therein]. The photon-nuclei interaction benefits from high nuclear densities in solids (~10^28^ m^−3^) and from a resonant cross section being about 400 times higher than the electronic scattering cross section at this energy, resulting in large optical thicknesses even in physically thin absorbers. Even at room temperature, nuclear resonances in solids exhibit ultrahigh quality factors and long coherence lifetimes, e.g., for ^57^Fe, *Q* ~10^13^ and τ_0_ = 141 ns, respectively. These are important prerequisites for quantum memories where phase and amplitude of the stored qubit must be preserved. Coherent temporal control of the excited nuclear quantum state was demonstrated, for example, by tuning the magnetic hyperfine interaction via magnon excitations (*14*) or via fast reversal or rotation of the nuclear magnetization (*11*, *15*–*17*), as well as by induced phase shifts via sudden movements of the resonant absorber (*18*–*21*), fast vibrating absorbers (*22*–*27*), thin-film x-ray cavities (*28*–*32*), or motion-induced Doppler shifts.

It is the latter approach that enables the formation of a quantum memory for hard x-ray photon wave packets using a set of moving resonant nuclear ^57^Fe absorbers (*12*). With the small transition linewidths and the typical high transition energies *E*_0_, a sizeable Doppler shift ∆*E* = *E*_0_*v*/*c*, *c* being the speed of light, is achieved at comparatively small velocities *v* (a velocity of 0.1 mm/s readily shifts the ^57^Fe transition by its natural linewidth). The proposed quantum memory concept is based on moving multiple resonant absorbers with different but equidistantly spaced velocities to form a Doppler nuclear frequency comb (NFC) in the resonant absorption structure. An incident resonant photon wave packet entering such a nuclear ensemble gets stored in the coherent collective nuclear excitation and is reemitted with a high probability at certain moments in time *T_k_* (*k* = 1,2,3, …), which are determined by the velocity spacing ∆*v* between neighboring absorbers (*12*),

$$T_{k}=k\frac{\mathrm{hc}}{E_{0}∆v}$$ (1)

where *h* is the Planck constant. Such periodic reemissions are referred to as “echoes.” With the frequency comb parameters (bandwidth, teeth width, and number of teeth) tuned correctly, an efficient and faithful quantum memory can be constructed, similar to atomic frequency combs in the optical regime (*33*–*35*). However, while atomic frequency combs are prepared by spectral hole burning within the broad inhomogeneous absorption linewidth by strong optical pulses that cannot be applied to nuclear systems, the NFC preparation is conceptually much simpler and straightforward. This promises to yield much sharper frequency combs that are ideally suited for quantum information storage.

Motivated by extending the quantum memory concept to the hard x-ray regime, we experimentally implement a robust NFC with up to seven teeth. After excitation with synchrotron x-ray pulses, the prepared NFC dramatically reshapes the temporal response of the coherent collective nuclear excitation of ^57^Fe nuclei in the forward scattering direction. This results in the formation of x-ray wave packets on the single-photon level with durations inverse to the frequency comb’s bandwidth. When adding a thin-film x-ray cavity, spectrally designed to match the frequency comb’s bandwidth, the wave packet of the emitted weak coherent field by the cavity is stored and retrieved by the NFC with a notable efficiency and fidelity. This storage of a wave packet on a single-photon level showcases the capability of the NFC to function as a quantum memory in the hard x-ray range.

## RESULTS

### Coherent resonant scattering from an NFC

**Fig. 1.**
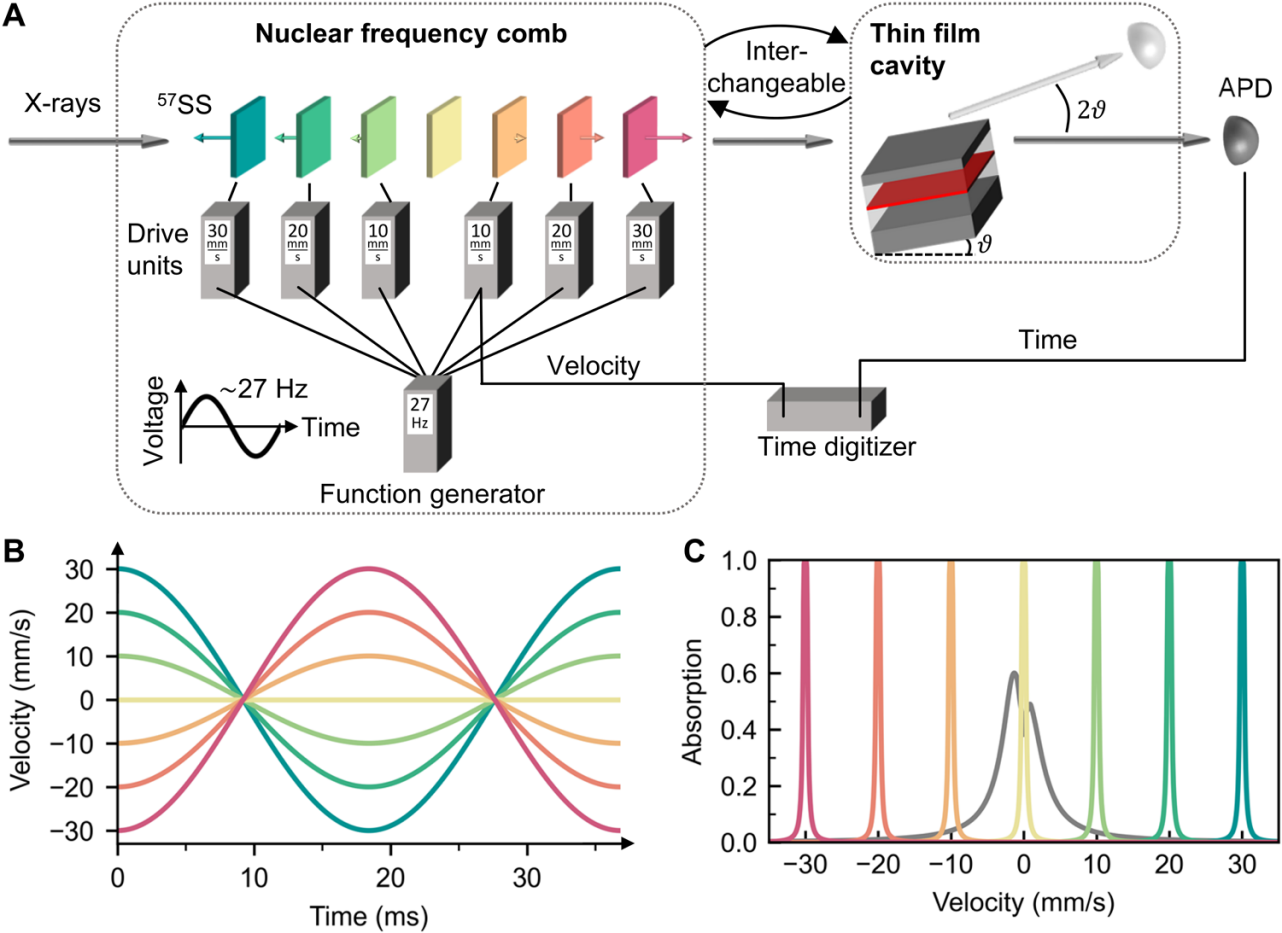
Experimental setup. (**A**) The NFC is formed by isotopically enriched 3.2-μm ^57^Fe stainless steel foils (^57^SS) mounted on Mössbauer transducers (not shown), each driven by a drive unit. One ^57^SS foil is kept at rest. Each drive unit amplifies the AC voltage generated by a single function generator differently such that the foils move with maximum velocities of 30, 20, 10, −10, −20, and −30 mm/s. The negative sign indicates a 180° motional phase shift realized by flipping the respective transducer. The foils are excited by 14.4-keV x-ray pulses from the synchrotron. The photon detection time and the instantaneous velocity of one Mössbauer transducer are simultaneously recorded using a multiple-event time digitizer. The cavity is mounted downstream but can conceptionally be viewed as upstream of the comb (see Materials and Methods). (**B**) Each foil’s velocity profile following the 27-Hz AC driving voltage. (**C**) Combined nuclear resonant absorption spectrum of the foils driven at maximum velocity, resulting in a frequency comb with a velocity spacing of 10 mm/s (= 480 neV). Spectrum of the photon wave packet emitted by the x-ray cavity is plotted as well (gray, arb. u.).

The setup contains seven single-absorption line isotopically enriched stainless steel foils (^57^Fe_0.55_Cr_0.25_Ni_0.2_) in a row with six of them mounted on Doppler velocity transducers, as sketched in Fig. 1A. The transducers are controlled by drive units synchronized with one sinusoidal master signal such that the foils’ motions follow the same sinusoidal pattern but with different maximum velocities. The maximum velocities are set equidistantly with a spacing of 10 mm/s, resulting in velocity profiles for each foil as shown in Fig. 1B. With the foil velocities following the master signal, the velocity spacings, and thus the Doppler shift between neighboring foils keep changing but remain always equidistant. For example, at maximum velocities, the Doppler-shifted foils form a frequency comb in the combined absorption spectrum with a velocity spacing of 10 mm/s (= 480 neV), as depicted in Fig. 1C.

The experiments were conducted at the High-Resolution Dynamics Beamline P01 at PETRA III (DESY, Hamburg, Germany) (*36*) and at the Nuclear Resonance Beamline ID18 at ESRF (Grenoble, France) (*37*). In the experiments, the temporally short (<100 ps) and spectrally broad (~0.5 meV or ~10,000 mm/s) synchrotron x-ray pulse resonantly excites the 14.4 keV transition of the ^57^Fe nuclei while propagating through all foils in the forward direction. Thereby, the nuclei are dynamically coupled to each other through the resonant field, and thus, the field and nuclei must be described together as a single coherent collective excitation state—a so-called nuclear polariton (*38*–*40*). In the presence of multiple transition frequencies in the nuclear medium, which in our case are caused by Doppler shifts, the subsequent decay of the collective excitation features pronounced temporal beat patterns in the forward scattering direction due to inter-resonance interferences. Note that, despite the brightness of modern synchrotron radiation, the average number of photons per pulse within the spectral range of the frequency comb is about 0.01. Therefore, the collective excitation almost never carries more than one photon. The decay of the nuclear polariton in the forward scattering direction is detected by a set of four stacked avalanche photon diodes (APDs), counting single-photon events as a function of arrival time relative to the x-ray pulse, whereby the x-ray pulse separation (192 ns at PETRA III; 176 ns at ESRF) exceeds the natural lifetime (141 ns for ^57^Fe). Collecting many of these single-photon decay events results in a histogram that represents the waveform of the wave packet emitted upon the radiative decay of the nuclear polariton or, synonymously, to the time-dependent photon emission probability of the nuclear polariton (*26*). Besides the arrival time, the instantaneous velocity spacing of the concomitant frequency comb is also recorded, rendering the histogram two-dimensional. With the foils’ velocity variations happening on timescales much slower (millisecond^−1^) than the nuclear decay rate (nanosecond^−1^), the velocities are virtually constant over the nuclear lifetime, resulting in a static frequency comb during each scattering process.

**Fig. 2.**
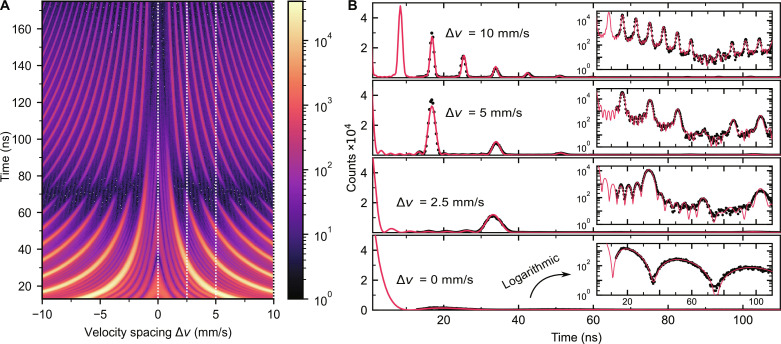
Coherent nuclear forward scattering from NFC. (**A**) Time-velocity histogram of the single-photon detection events resulting from the decay of a nuclear polariton spectrally prepared in a frequency comb consisting of seven teeth, equally spaced over a Doppler detuning range extending from −10 to 10 mm/s (≈ 480 neV). The color map displays the number of detected photons on a logarithmic scale. (**B**) Decay histogram for four selected velocity spacings from (A), indicated by dashed lines, in linear and logarithmic scales. The patterns are simulated using the software package Nexus (red).

The accumulated histogram for the frequency comb setup without x-ray cavity is shown in Fig. 2A, from which four columns corresponding to four different velocity spacings between the foils are selected in Fig. 2B. The histograms in Fig. 2B reflect the unnormalized photon emission probability of the nuclear polariton for different velocity spacings and clearly show equidistantly repeating time instants of enhanced photon emission—the echoes from the frequency comb. From Eq. 1, the echo period increases from ∆*T* = 8.6 to 34 ns when the velocity spacing reduces from ∆*v* = 10 to 2.5 mm/s, agreeing well with the experimental observations. In the meantime, the full width at half maximum of the echoes decreases from 4.4(3) ns at 2.5 mm/s to 1.2(1) ns at 10 mm/s, showing an inverse proportionality to the total bandwidth of the frequency comb. The observed temporal patterns closely resemble spatial interference patterns obtained from diffraction gratings (see the Supplementary Materials).

The drastic reshaping of the emission probability due to the frequency comb is best seen in comparison with the case when no foil is moving (∆*v* = 0 mm/s). In this case, the emission probability follows the superradiant decay from a 22.4-μm-thick stainless steel foil (7 × 3.2 μm) with dynamical beats typical for optically thick absorbers due to multiscattering (*12*, *40*). However, when the foils are moving and forming a frequency comb, the emission probability is completely reshaped into a repetitive structure, where the emission is drastically enhanced at the echoes and decreased in between.

The experimental result is supported by simulations carried out using the software package Nexus (*41*), which calculates the total (resonant and nonresonant) forward scattering intensity of the experiment in frequency domain (*42*). Both agree with our time-domain simulations based on Maxwell-Bloch equations. With material and hyperfine parameters of each foil being determined by previous characterization (see the Supplementary Materials), the simulations shown in Fig. 2B match the measurements very well, which demonstrates the accurateness of our setup. In addition, the predicted temporal beat patterns in between two echoes, arising because of partial constructive interferences between the Doppler-shifted transitions, are experimentally well resolved.

### Quantum storage of a hard x-ray photon wave packet

**Fig. 3.**
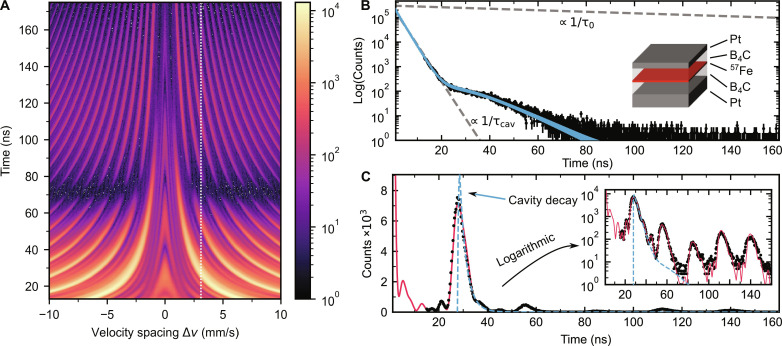
Quantum memory for hard x-ray photon wave packets. (**A**) Time-velocity histogram of the same frequency comb as shown in Fig. 2, in combination with a thin-film x-ray cavity excited in its first waveguide mode at an incident angle of 0.1505°. (**B**) Measured (black) and fitted (blue) decay histograms of the thin-film cavity alone, excited in its first waveguide mode. The initial strong speed-up in the cavity decay corresponds to an exponential decay [∝ exp(−*t*/τ_cav_), with τ_cav_ = 2.9(2) ns], in comparison to the natural exponential decay of the excited ^57^Fe state with τ_0_ = 141 ns. The inset shows the layer structure of the cavity consisting of: Pt (2.3 nm)/ B_4_C (13.1 nm)/ ^57^Fe (1.1 nm)/ B_4_C (12.8 nm)/ Pt (15.0 nm)/ Al_2_O_3_ (substrate; not shown). (**C**) Extracted decay histogram from (A), indicated by dashed line, at the velocity spacing of 3.1 mm/s (≈ 240 neV) with measurement (black) and fit (red) in linear and logarithmic scales. The derived x-ray cavity decay from (B) is overlaid (blue) at the first echo, showing the matching decay of the echo and the cavity intensities. More decay histograms for different velocity spacings are depicted in the Supplementary Materials.

The results confirm a well-operating NFC. To evaluate the performance of the NFC as a quantum memory, a wave packet on a single-photon level with a spectrum matching the comb bandwidth is required rather than the spectrally broad synchrotron pulse. For that purpose, we used a thin-film x-ray cavity. The cavity consists of a stack of nanometer thin films (see sketch in Fig. 3B; details in Materials and Methods and the Supplementary Materials). At a particular incidence angle, the x-ray field couples into the first-order waveguide mode and peaks in the center of the cavity, where an isotopically enriched ^57^Fe layer is located. This enhances the photon-nuclei interaction, resulting in a superradiant collective nuclear state (*29*). Accordingly, the measured waveform of the subsequently emitted wave packet, depicted in Fig. 3B, shows an accelerated decay: In the first 20 ns, the decay follows an exponential law with a drastically reduced lifetime of τ_cav_ = 2.9(2) ns. This corresponds to a transition linewidth being 47(3) times broader than the natural linewidth Γ_0_ of the ^57^Fe resonance (4.7 neV) and, thus, fitting within the bandwidth range of the frequency comb; see Fig. 1C. The fast decay is slowed down after 20 ns when a beating sets in, caused by a residual quadrupole splitting of the nuclear levels of 29 neV. This splitting is much smaller than the superradiant broadening, and its effect on the spectral shape of the emitted wave packet is negligible.

This wave packet emitted by the x-ray cavity corresponds to a weak coherent state on a single-photon level with an average photon number of about 0.01. While it is not a true single-photon state, the weak coherent state on the single-photon level is a common tool to test efficiency and fidelity of quantum memories (*5*).

In our experiment, an x-ray cavity was mounted downstream of the seven foils and aligned in a grazing incidence configuration along the x-ray beam path as depicted in Fig. 1A. This seemingly counterintuitive sequence was chosen for technical simplification of the setup. However, conceptionally, the x-ray cavity can be viewed as upstream. With nuclear forward scattering being an inherently coherent and, in our case, linear scattering process, the whole setup can be treated as one single device where regardless of the specific sequence along the scattering path, the outcome remains always the same (see Materials and Methods).

The collected histogram of the combined cavity-frequency comb setup is shown in Fig. 3A. Again, enhanced emission at the echoes is clearly visible, but the echo shape becomes temporally asymmetric (a direct comparison between Figs. 2A and 3A is depicted in fig. S6). The photon emission for a velocity spacing of 3.1 mm/s, shown in Fig. 3C, fully confirms the asymmetric shape: The rising edges of the echoes are very sharp, while the falling edges follow an exponential decay (linear slope in the logarithmic representation). The echo shape exactly reflects the photon wave packet shape emitted by the x-ray cavity alone. It is both visible in the experimental data and in the simulations. In other words, the weak coherent wave packet emitted by the cavity is stored and retrieved at times defined by the velocity spacing, which largely exceed the wave packet’s duration, characterized by τ_cav_. This demonstrates the functionality of the NFC as a quantum memory.

The memory’s performance can be quantified by the echo efficiency, i.e., the number of emitted photons within an echo duration relative to the total number of coherently forward-scattered photons, and by the fidelity of the storage process, i.e., how well the temporal shape of the wave packet emitted by the cavity is preserved (see calculation details in Materials and Methods). With the simulations matching the measurements very well, they can be justifiably used to extrapolate the temporal patterns to time zero. Experimentally, it is not accessible because of a strong nonresonant part of the synchrotron pulse perturbing the detection scheme at early times. The extrapolation enables the evaluation of the total number of emitted photons and, therefore, of the echo efficiency and fidelity. Because of the lack of phase information in our measurements, only a photon counting–based fidelity is calculable. Because nuclear forward scattering is inherently coherent, for static NFCs, the phases of the echoes are predicted to be well preserved, rendering the intensity fidelity a good approximation for the complex amplitude-based fidelity (*12*, *43*).

**Fig. 4.**
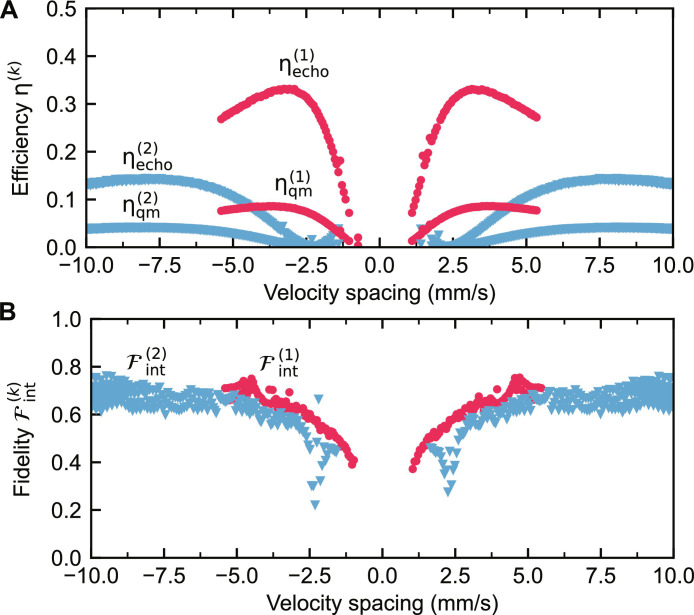
Quantum memory parameters. Derived echo efficiency $\eta_{\text{echo}}^{\left(k\right)}$ , quantum memory efficiency $\eta_{\text{qm}}^{\left(k\right)}$ (**A**), and intensity fidelity ${ℱ}_{\text{int}}^{\left(k\right)}$ (**B**) for the *k*th echo [*k* = 1 (red) or *k* = 2 (blue)] of the cavity-frequency comb setup as a function of the velocity spacing. For echoes appearing early, <13 ns (detection dead time), or late, at >100 ns (low intensity), no meaningful values of efficiency and fidelity could be derived.

The calculated echo efficiency as a function of the velocity spacing is shown in Fig. 4A. The echo efficiency peaks for the first echo at 33(1)% for a velocity of 3.1 mm/s and decreases for low-velocity spacings quickly while for high-velocity spacings more moderately. This is mainly caused by two effects: first, relevant for small velocity spacings, by the lifetime of the nuclear polariton, which is related to the spectral width of a single comb tooth and fundamentally limited by the natural lifetime of the ^57^Fe resonance (141 ns), and, second, by the decay into higher-order echoes, relevant for high-velocity spacings.

Note that the calculated echo efficiency does not account for photon losses due to nonresonant electronic charge interactions and incoherent nuclear scattering, which result in a total energy loss of 74% for a velocity spacing of 3.1 mm/s (see the Supplementary Materials). A quantum memory efficiency accounting for these losses is depicted in Fig. 4A. For 3.1 mm/s, η_qm_ is 8% for the first echo, a value comparable to optical fixed-delay atomic frequency combs (*44*–*47*). This can be compared to the analytical quantum memory efficiency as derived for the storage of a Gaussian wave packet (*12*). For the same experimental parameters and including electronic interactions, it yields 13.1%, with a theoretical upper limit of 18.4% (54% if electronic interactions are neglected) imposed by multiscattering (*12*). Despite the different stored wave packet shapes, the measured efficiency is close to the theoretical predictions and not far from the upper limit. An efficiency increase could be accomplished by increasing the optical thickness of the foils (*12*). Furthermore, higher efficiencies could also be realized by more sophisticated nuclear quantum memory protocols, which require the use of fast piezoelectric materials (*12*).

The calculated fidelity is shown in Fig. 4B. It monotonically increases with the velocity spacing but saturates for higher velocities at 67(4)%. At very low-velocity spacings (≲1.5 mm/s), the comb bandwidth is too narrow to maintain the temporal waveform of the cavity decay. Moreover, the echo shape can be altered by the single-foil dynamical beat node at around 70 ns as they temporally coincide at ∼1.25 and ∼2.5 mm/s for the first and the second echoes, respectively. For large velocity spacings, the number of measurement points becomes insufficient for a precise calculation of the fidelity due to the reduced echo period, noticeable by the increased spread of fidelity values in Fig. 4B.

The fidelity of the photon emission does not quite reach values necessary for faithful quantum memory operation (typically >90%). This is largely due to the step-like rising edge in the cavity decay at time zero. If this edge is neglected, the derived fidelity reaches 97(1)%. Such a step-like edge cannot be well reproduced by the frequency comb because of its limited bandwidth. The comb bandwidth can be increased by adding more foils while keeping the same velocity spacing (a comb with 11 teeth would result in a fidelity of 75%). In addition, an “imperfect source,” i.e., nonresonantly scattered cavity photons, also contributes to the fidelity degradation (see Materials and Methods). The fidelity can be substantially increased for photon wave packets with a smooth leading edge, which can be produced by passing the radiation from a Mössbauer synchrotron source through a vibrating resonant absorber (*26*).

In total, for the given optical thicknesses of the foils and cavity decay properties, a velocity spacing of 3.1 mm/s appears to be optimal for storing and recovering the wave packet emitted by the cavity with good efficiency and decent fidelity.

## DISCUSSION

The set of Doppler-shifted ^57^Fe absorbers demonstrates a well-operating NFC. It allows for drastically reshaping the emission characteristics of the nuclear polariton to produce strong and short echo signals well above the noise level. In combination with the x-ray thin-film cavity, the NFC shows that it is capable of storing and retrieving wave packets of a weak coherent field on a single-photon level with notable efficiency and fidelity. The achieved storage time of about 30 ns exceeds the wave packets duration by an order of magnitude. The demonstrated ability to work on a single-photon level is a common benchmark for quantum memory protocols (*5*) and shows that the NFC constitutes a quantum memory in the x-ray regime. The NFC works conceptually identical to the atomic frequency comb protocols (*33*, *35*).

The demonstrated NFC protocol can be applied to produce time-bin waveforms at x-ray energies as it splits an incoming photon wave packet into temporally separated copies (*12*). Advancing the NFC further, a fast and versatile control over the echo emission can be achieved by substituting the Mössbauer transducer with piezoelectrical materials, which allows alteration of the absorption structure during the nuclear lifetime (*12*). Moreover, integrating the NFC with a nondeterministic single-photon source could enable an on-demand single-photon source (*1*, *12*). For example, this could be realized by using the radioactive decay cascade of ^57^Co to herald a 14.4-keV single photon (*22*).

The NFC is not restricted to the ^57^Fe resonance but can be applied to all Mössbauer nuclear resonances, including the long-lived isomer transitions in ^45^Sc at 12.4 keV or in ^229m^Th at 8.3 eV, which are both promising candidates for nuclear clocks (*48*–*50*). With the effective broadening of the absorption spectrum and its distinct echo structure, the NFC can be a tool for enhancing the excitation and detection efficiency of the extremally narrow resonances of 0.1 Hz (^45^Sc) and 0.1 mHz (^229m^Th) while still using the existing orders of magnitude broader radiation sources. Because of the long lifetime, even a spatial separation of the echo signal from the nonresonant background is possible by displacing the whole NFC during the storage process, which would further increase the detection efficiency.

## MATERIALS AND METHODS

### Experimental details on the frequency comb setup

The stainless steel (^57^Fe_0.55_Cr_0.25_Ni_0.2_ wt %) foils were produced by HMW Hauner GmbH&Co. KG, whereby the iron is isotopically enriched in ^57^Fe to 95%. The thickness, 3.21(5) μm, was revealed from the measurement of the nuclear decay of a single foil, which also provides the foil’s hyperfine parameters (details are given in the Supplementary Materials). The Mössbauer transducers (Wissel MVT-1000 and MA-260S as well as FAST ComTec MR-250) are of a double-loudspeaker type, where one coil drives the mounted sample (in our case, the stainless steel foil) and the other coil picks up the motion and converts it to a voltage signal so that a feedback can be provided to a control loop mechanism, which is executed by the Mössbauer drive unit (Wissel MR-360 and MDU-1200 as well as FAST ComTec GmbH MR-351). This ensures that the sample follows the desired velocity pattern within an error of <0.1%. For the highest velocity used, 30 mm/s, the error is about 0.03 mm/s, which is smaller than the natural linewidth of the ^57^Fe transition (0.097 mm/s). To establish a nearly perfect synchronization, only one master frequency generator (the built-in generator of the drive unit MR-351) was used to provide the input signal for all driving units. The frequency was set to 27.1(2) Hz, which roughly coincides with the resonant frequencies of the used transducers (between 22 and 27.5 Hz).

To calibrate the velocities of each transducer, a two-dimensional histogram was taken where the stainless steel foil mounted on a single moving transducer was measured versus a magnetized ^57^Fe foil whose absorption line is hyperfine-split into six lines due to its internal magnetic hyperfine field of 33 T. Since the hyperfine splitting of the six lines is well known and used as an energy reference in Mössbauer spectroscopy, the exact velocity of the transducer can be derived from the interference between the Doppler-shifted stainless steel foil and the six reference lines. In total, the maximum velocity could be set with a precision of better than 1% of the desired velocity. Even after physically moving the transducer several times in and out of the setup, a calibration recheck provided a nearly identical velocity (deviation <0.01 mm/s).

The two-dimensional histograms were obtained by a multi-event time digitizer (MCS6A by FAST ComTec GmbH) synchronized to the synchrotron bunch clock. It records the arrival time of the resonantly scattered photons, measured by a stack of four APDs, and the velocity from the connected Mössbauer drive unit. The digitizer’s temporal resolution is 0.1 ns, while the APD’s resolution is about 0.5 ns. The sampling of the sinusoidal drive signal by 1024 channels is equidistant in time, meaning that velocities are sampled finer around high velocities and coarser at low velocities.

### Experimental details on the cavity structure and setup

The thin-film cavity consists of the layer system (see sketch in Fig. 3B):

Al_2_O_3_ (substrate) / Pt (15.0 nm) / B_4_C (12.8 nm) / ^57^Fe (1.1 nm) / B_4_C (13.1 nm) / Pt (2.3 nm).

It was fabricated in our laboratories via magnetron sputtering in a high-vacuum chamber with a base pressure <6 × 10^−7^ mbar. The sputter targets of the used materials have a purity higher than 99.5%, and the iron target is enriched in ^57^Fe to 95%. Under an argon atmosphere of 5 × 10^−3^ mbar, the layers were deposited sequentially on the sapphire substrate using DC power (for the metal materials) or radiofrequency power (for boron carbide). The cavity was characterized at the synchrotron by the nonresonant and nuclear resonant reflectivity of the sample, providing information about the thicknesses and roughnesses of each layer, as well as by the temporal beat pattern after x-ray excitation to gather information about the hyperfine parameters and the superradiant broadening in the first-order cavity minimum. The iron layer shows no indication of a magnetization, which is due to its low thickness that prevents the formation of long-range magnetic order (*29*). More details on the characterization measurements are given in the Supplementary Materials.

The cavity was mounted on a goniometer to align the sample with an angular precision of 0.0005°. The x-rays at the synchrotron were focused by a Be compound refractive lens to a beam size at the sample position of 70 μm (vertical) by 310 μm (horizontal). A vertical focus is beneficial since the experiments were conducted at small incident angles (about 0.15°) where the footprint of the unfocused beam (height ≈0.7 mm) would be much longer than the reflecting sample (15 mm). The focusing produces a beam divergence of about 0.003°.

### Sequence of frequency comb and cavity

To facilitate a substantial technical simplification of the setup, the thin-film cavity was mounted downstream of the foils. While this seems contradicting with the claim of the paper that the wave packet emitted by the cavity is stored in the frequency comb, it is important to note that the nuclear forward scattering process is coherent and linear in our setup. The coherence is inherently given when detecting delayed photons in the nuclear forward scattering direction, which is only possible if the scattering process was coherent (*38*, *39*). In addition, nonlinear effects are not present in nuclear forward scattering of synchrotron radiation due to the excitation being very weak (mean resonant photon number around 0.01). Despite that, in general, the sequence of absorbers in a nuclear resonance scattering experiment can be important but only if the temporal evolution is perturbed on the timescale of the radiative nuclear lifetime (*51*, *52*). Since, in our case, the foils can be well approximated to be moving with constant velocity during the scattering process, the whole setup is known to be commutative (*52*). Thus, whether the cavity is mounted down- or upstream (or even in between the foils) does not influence the outcome of the scattering process. Therefore, the cavity can always be interpreted to be upstream of the foils, rendering this the “conceptual” setup.

### Calculation of the efficiency

The quantum memory efficiency of the *k*th echo, $\eta_{\text{qm}}^{\left(k\right)}$ , can be translated into photon number quantities in a straight-forward way via (*12*),

$$\eta_{\text{qm}}^{\left(k\right)}=\frac{1}{N_{\text{tot}}}\int_{T_{k}-\text{FWHM}}^{T_{k+1}-\text{FWHM}}n_{\text{meas}}\left(t\right)\mathrm{dt}$$ (2)

with *T_k_* being the time of the *k*th echo appearance, FWHM being the full width half maximum of the echo, which is approximately the same for all echoes (deviation < 0.3 ns) for a fixed velocity spacing, and *n*_meas_(*t*) being the measured delayed photon counts from the decay of the combined setup (cavity plus NFC) as displayed in Fig. 3A. *N*_tot_ is defined as the total number of incoming photons. However, this quantity is not experimentally accessible.

On the other hand, another efficiency, called echo efficiency $\eta_{\text{echo}}^{\left(k\right)}$ , can be directly derived. This echo efficiency is the probability that a resonantly forward-scattered photon appears at the *k*th echo. Consequently, the normalization constant for this efficiency $\eta_{\text{echo}}^{\left(k\right)}$ is the total number of resonantly forward-scattered photons, $N_{\text{FS}}=\int_{0}^{t_{\text{end}}}n_{\text{meas}}\left(t\right)\mathrm{dt}$ , with the upper integration bound, *t*_end_, given by the separation of two incident x-ray pulses (192 ns at PETRA III and 176 ns at ESRF). In the experiment, the large nonresonant component of the pulse renders a high background at time zero at the detectors, which leads to artefacts in the detection scheme up to several nanoseconds after the x-ray pulse passes. Therefore, a time gating is implemented, which prevents counting of resonantly scattered photons before 13 ns. To account for the missing intensity in the calculation of the normalization constant *N*_FS_, the collected histogram was extrapolated to time zero by simulating the resonant nuclear response with Nexus (*41*). The good agreement of the simulated temporal beat pattern with the measured decay histogram, as shown in Fig. 3C, justifies this approach.

The two normalization constants, *N*_FS_ and *N*_tot_, are related to each other via

$$N_{\text{FS}}=\left(1-\beta_{\text{el}}-\beta_{\text{nis}}\right)N_{\text{tot}}=\left(1-\beta_{\text{tot}}\right)N_{\text{tot}}$$ (3)

where β_el_ and β_nis_ are the photon losses due to nonresonant electronic charge interactions (Compton scattering and photoelectric absorption) and nuclear incoherent scattering (inelastic phonon excitations, internal conversion, and nuclear fluorescence), respectively. These losses are evaluated to β_tot_ = 74% (see the Supplementary Materials). By calculating *N*_tot_ with Eq. 3, the quantum memory efficiency $\eta_{\text{qm}}^{\left(k\right)}$ as defined in Eq. 2 can be derived, which, for the optimal velocity spacing of 3.1 mm/s, is 8% for the comb-cavity setup.

Besides the intrinsic loss channels, there are also losses due to the setup. The four stacked APDs have a combined single-photon detection efficiency of 0.65, meaning that 65% of the photons reaching the detector are counted. Moreover, there is a probability that a resonant photon interacts with the frequency comb foils but not with the cavity. This “source efficiency” depends on the cavity’s scattering factor, which is strongly energy dependent. At exact resonance, the maximum interaction probability is reached at 77(1)% but decreases to an average value of 55(1)% within the spectral interval covering three times the superradiantly broadened resonance linewidth of the cavity mode (3Γ_cav_ = 3 · 47Γ_0_ = 663 neV). In contrast, the probability that a resonant photon does not interact with the frequency comb is nearly zero due to the large optical thickness of the foils.

These systematic loss channels of the setup are not intrinsic properties of the NFC, and therefore, are not accounted for in the reported quantum memory efficiency $\eta_{\text{qm}}^{\left(k\right)}$.

### Calculation of the fidelity

In contrast to the efficiency, the fidelity cannot be easily translated into a photon number quantity. However, an upper boundary can be estimated, which is nonetheless close to the complex amplitude-based fidelity for static frequency combs as used in this work (*12*, *43*). The complex amplitude-based fidelity of the *k*th echo is defined in (*12*, *53*)

$${ℱ}^{\left(k\right)}=\frac{1}{N_{\text{cav}}N_{\text{meas}}}{\left|\int_{T_{k}-\text{FWHM}}^{T_{k+1}-\text{FWHM}}{ℇ}_{\text{cav}}^{†}\left(t-T_{k}\right){ℇ}_{\text{out}}\left(t\right)\mathrm{dt}\right|}^{2}$$ (4)

where ℇ_cav_(*t*) and ℇ_out_(*t*) are the complex wave packet amplitudes of the photon decay from the cavity mode and from the combined setup (cavity plus NFC), respectively. The normalization constants are given by: $N_{\text{cav}}=\int_{T_{k}-\text{FWHM}}^{T_{k+1}-\text{FWHM}}{\left|{ℇ}_{\text{cav}}\left(t\right)\right|}^{2}dt$ and $N_{\text{meas}}=\int_{T_{k}-\text{FWHM}}^{T_{k+1}-\text{FWHM}}{\left|{ℇ}_{\text{out}}\left(t\right)\right|}^{2}\mathrm{dt}$ . In our experiment, only the number of emitted photons is accessible (see Fig. 3), which is proportional to *n*_cav_(*t*) ∝ ∣ℇ_cav_(*t*)∣² and *n*_meas_(*t*) ∝ ∣ℇ_out_(*t*)∣². An inequality can be derived to estimate the upper boundary of the fidelity

$$\begin{array}{c} {ℱ}^{\left(k\right)}\leq \frac{1}{N_{\text{cav}}N_{\text{meas}}}{\left[\int_{T_{k}-\text{FWHM}}^{T_{k+1}-\text{FWHM}}|{ℇ}_{\text{cav}}^{†}\left(t-T_{k}\right){ℇ}_{\text{out}}\left(t\right)|\mathrm{dt}\right]}^{2}=\\ \frac{1}{N_{\text{cav}}N_{\text{meas}}}{\left[\int_{T_{k}-\text{FWHM}}^{T_{k+1}-\text{FWHM}}\sqrt{n_{\text{cav}}\left(t-T_{k}\right)n_{\text{meas}}\left(t\right)}\mathrm{dt}\right]}^{2}≝{ℱ}_{\text{int}}^{\left(k\right)} \end{array}$$ (5)

This intensity fidelity (*43*), ${ℱ}_{\text{int}}^{\left(k\right)}$ , is plotted in Fig. 4. Note that for a well-defined fidelity, the echo spacing Δ*T* = *T*_*k*+1_ − *T_k_* must be bigger than the cavity decay constant. Here, the cavity decay constant is 2.9(1) ns. Thus, the assumption is true for all measured velocity spacings (Δ*v*_max_ = 10 mm/s $\hat{=}\Delta T_{\text{min}}=8.6$ ns).

Imperfect source efficiency, i.e., not all photons interacted resonantly with the cavity, can strongly affect the fidelity as calculated above, which assumes a perfect cavity source. For example, if barely any photon had interacted with the cavity, the genuine source seen by the NFC would be completely different so that the calculated fidelity would be substantially lower than the factual value, although the intrinsic nuclear resonant scattering properties of the NFC had not changed. Thus, in case of an imperfect source, as present in this work, the derived fidelity value cannot reach unity.

## Supplementary Materials

### This PDF file includes:

Supplementary Text Figs. S1 to S6 Tables S1 to S3 References
