# Supplementary material for: Nuclear quantum memory for hard x-ray photon wave packets
Source: Sci Adv. 2024 Jun 26;10(26):eadn9825. doi: 10.1126/sciadv.adn9825 (PMC11204287; doi:10.1126/sciadv.adn9825)
Supplement: Supplementary file 1 — Supplementary Text Figs. S1 to S6 Tables S1 to S3 References [file sciadv.adn9825_sm.pdf]

Science Advances
AAAS

Supplementary Materials for

Nuclear quantum memory for hard x-ray photon wave packets

Sven Velten *et al.*

Corresponding author: Sven Velten, sven.velten@desy.de; Ralf Röhlsberger, r.roehlsberger@gsi.de

Sci. Adv. **10**, eadn9825 (2024)
DOI: 10.1126/sciadv.adn9825

This PDF file includes:

Supplementary Text
Figs. S1 to S6
Tables S1 to S3
References

Supplementary Text

In the following more numerical and experimental details, as well as additional measurements, are given to support the conclusions drawn in the main text.

Data fitting

At multiple points, calculations and least-square fits were numerically conducted by the software package *Nexus* to derive certain physical parameters. The fit routine relies on either the Levenberg-Marquardt or the Differential Evolution algorithm. Both algorithms minimize a squared residual, which is calculated for each data point i from the residual,

$$\mathrm{res}_i = \sqrt{y_i} - \sqrt{y_i^s}, \tag{S1}$$

where y and y^s refer to the experimental and simulated data, respectively. The actual cost function, which is minimized, is half of the sum over all squared residuals,

$$\mathrm{cost} = \frac{1}{2}\sum_i \mathrm{res}_i^2. \tag{S2}$$

For further information, readers are referred to the documentation of *Nexus* (*41*).

Nuclear resonance scattering of a single stainless-steel foil

The decay histogram of one of the stainless-steel foils is shown in Fig. S1. A dynamical beat node near 70 ns is clearly visible, caused by multiscattering effects in optically thick resonant absorbers. The decay pattern can be fit by varying the foil's thickness and the full width at half maximum (FWHM) of an assumed Gaussian broadened absorption line. The origin of the broadening is not further investigated since a small broadening has no effect on the measurements. Possible sources include inhomogeneous broadening, small thickness variations and small hyperfine splittings due

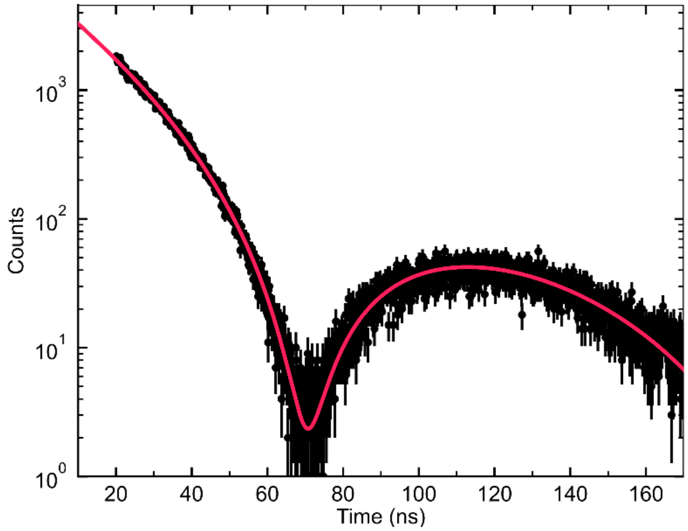

Fig. S1. Nuclear decay of single stainless-steel foil. Measured (black) and fitted (red) nuclear decay of one of the used stainless-steel ($^{57}Fe_{0.55}Cr_{0.25}Ni_{0.2}$ wt.%) foils. The fit corresponds to a 3.2 µm foil thickness which corresponds to an optical thickness of 27.

Table S1. Results of stainless-steel foils characterization. Fitted foil thicknesses and hyperfine parameters for each stainless-steel foil. The mean values are also given together with their standard deviation. For energy conversion: 0.097 mm/s = $1\Gamma_0$ = 4.66 neV.

Foil #	Thickness (µm)	Linewidth FWHM (mm/s)	Optical thickness
1	3.26	0.25	27.6
2	3.28	0.25	27.8
3	3.25	0.25	27.4
4	3.17	0.25	26.8
5	3.19	0.25	27.0
6	3.16	0.26	26.7
7	3.16	0.25	26.7
Average	3.21(5)	0.25(0)	27.1(4)

to residual magnetic fields or electric field gradients. From the fitted foil thickness an optical thickness ζ (also called effective thickness for nuclear resonance scattering) can be calculated, defined by (*40*),

$$\zeta = \sigma_0 f_{LM} \rho_n d, \tag{S3}$$

with σ_0= 2557.67 kbarn being the nuclear resonant cross-section, f_{LM}= 0.76 the Lamb-Mössbauer factor, ρ_n= $4.35\cdot10^{28}$ m^{-3} the number density of the resonant isotope ^{57}Fe and d the derived foil thickness. The optical thickness is the relevant quantity to describe nuclear resonant interactions as it includes the elastic-to-inelastic scattering ratio (Lamb-Mössbauer factor), the strength of coherent scattering (number density and foil thickness) and the internal conversion process [included in the nuclear resonant cross-section (*40*)]. The parameters for all foils as well as the mean values are given in Table S1. The small standard deviations of the mean values show that the foils only differ little. Therefore, the absorption line shapes of the frequency comb are nearly identical, which would otherwise undermine the comb's efficiency.

Thin film cavity characterization

In the grazing incidence setup at the synchrotron, the reflected intensities of the non-resonant part (X-ray reflectometry) and the resonant, delayed part (nuclear X-ray reflectometry) of the incoming 14.41 keV X-ray pulse were detected as a function of the incidence angle. While the former gives information about the electron density distribution inside the thin film cavity, the latter accesses the distribution of the resonant nuclei inside the sample. Together, the thickness and roughness of each layer can be derived by using a transfer matrix approach (*54*) (implemented in the software package *Nexus*). The experimental data and the calculated intensity are shown in Fig. S2A and B. The derived layer parameters are listed in Table S2. The hyperfine parameters can be obtained from the decay histogram, which is shown in Fig. S2C for two different incident angles: 0.1505° (the first order waveguide mode) and 0.45°. The fit results are listed in Table S3. For the quadrupole splitting, a Gaussian distribution with only positive values is assumed. To cross-check the derived parameters, the cavity setup is combined with a stainless-steel foil mounted on a Mössbauer transducer. A two-dimensional histogram, similar to the frequency comb measurement, containing information in both time and energy domain can be recorded by Doppler shifting the relatively narrow absorption line of the stainless-steel foil. This interferometric setup is reported to be able to access the energy spectrum of the cavity, if the cavity is illuminated at the critical coupling

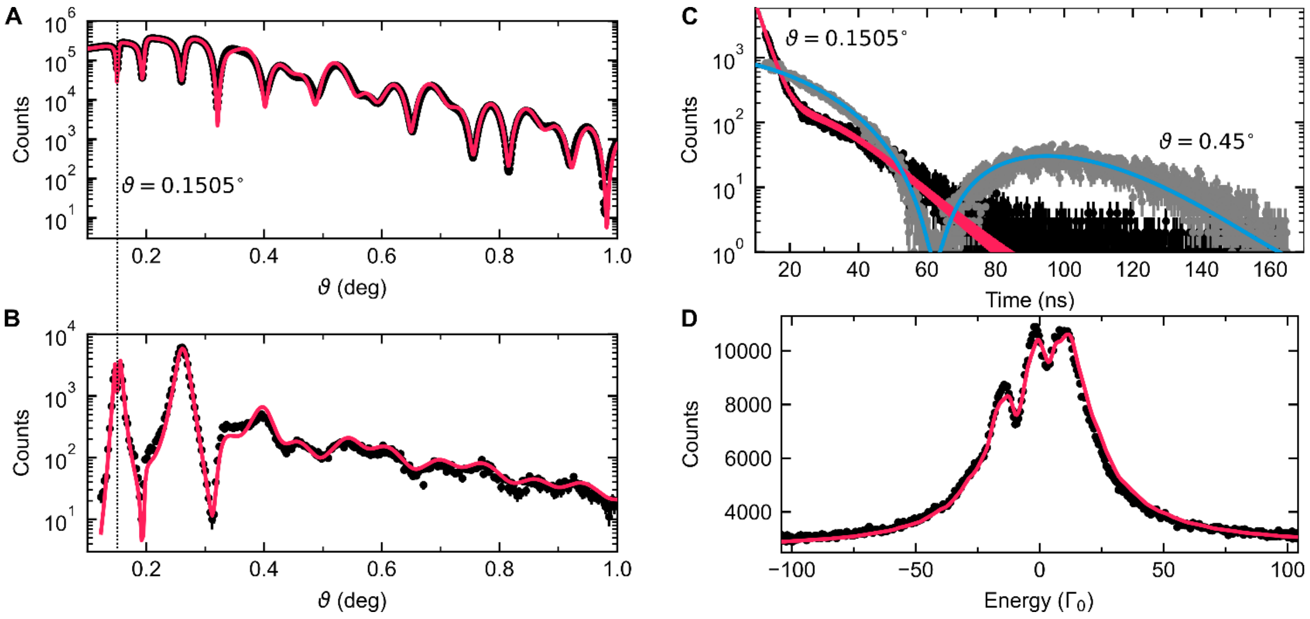

Fig. S2. X-ray cavity characterization measurements. Measurements on the thin film cavity. (**A**) Reflected intensity with only the non-resonant (non-delayed) photons counted. (**B**) Reflected intensity with only the resonant (delayed by >13 ns) photons counted. (**C**) Temporal beat pattern at the incidence angle 0.1505°, where the first order waveguide mode is excited [marked in (A) and (B) as well], and at 0.45°. (**D**) Energy spectrum at the incidence angle 0.1505° of the combined setup with a stainless-steel foil mounted on a Mössbauer transducer, obtained by an integration in the time window from 69 ns to 165 ns. Theoretical curves (red and blue solid lines) are either the numerical fits (A, C) or simulations (B, D) with parameters obtained from the other fits.

condition of a cavity minimum and the two-dimensional histogram is time integrated in a late time window (*29*). This is shown in Fig. S2D, where the integration time window was set between 69 ns and 165 ns. The shape and width of the calculated spectrum match the experimental data very well. For a good match, an isomer shift of 0.3 mm/s relative to the stainless-steel absorber must be assumed. The deviation from the known isomer shift between α-Fe and stainless-steel of 0.1 mm/s (*55*), is likely caused by the disordered nature of the ^{57}Fe atoms in the ultrathin iron layer consisting of only three to four atomic layers.

Influence of the number of foils in the frequency comb

In the main text, the frequency comb consists of seven ^{57}SS foils, i.e. seven teeth. To investigate the influence of the teeth number N on the frequency comb, stainless-steel foils were removed or added at the end of the comb, so that the remaining teeth stay always spectrally equidistant. In Fig. S3A, the temporal responses for a fixed velocity spacing of 5 mm/s are shown for frequency combs consisting of five to eight foils. Several trends are clearly visible as more comb teeth are introduced:

Table S2. Results of X-ray cavity characterization. Cavity layer structure derived from the non-resonant reflectivity measurement in Fig. S2A.

Layer material	Thickness (nm)	Density (g cm^{-3})	Roughness (nm)
Pt	2.3	21.4	0.4
B_4C	13.1	2.5	0.3
^{57}Fe	1.1	8.0	0.5
B_4C	12.8	2.4	0.0
Pt	15.0	19.3	0.4
Al_2O_3 wafer	-	3.98	0.0

Table S3. Hyperfine parameters of X-ray cavity. Incidence angle, divergence and hyperfine parameters derived from the temporal beat pattern in Fig. S2B and the energy spectrum in Fig. S2D. The isomer shift is relative to the stainless-steel foil used as a reference absorber. For energy conversion: 0.097 mm/s = $1\Gamma_0$ = 4.66 neV.

Data	Parameter	Fit
Temporal beat pattern in Fig. S2B	Incidence angle	0.1505° / 0.45°
	Divergence	0.0027°
	Quadrupole splitting	0.61 mm s^{-1}
	Quadrupole splitting FWHM	0.92 mm s^{-1}
Energy spectrum in Fig. S2D	Incidence angle	0.1502°
	Divergence	0.0037°
	Isomer shift	0.3 mm s^{-1}

the echoes get sharper in time, the intensity contrast between the echoes and their side maxima rises and the number of these side maxima increases by one with every newly added foil. In total, there are always $N-2$ side maxima and $N-1$ side minima. The observed characteristics strongly resemble diffraction intensity patterns known from spatial gratings. Here, however, the frequency comb constitutes a (single-photon) grating in the frequency domain. Translating the known diffraction intensity distribution from spatial gratings into a frequency grating by adding up N complex plane waves with different but equidistant frequencies, the intensity of the emitted photon wave packet should follow,

$$I(t) \propto \frac{\sin^2(N\Delta\omega t)}{\sin^2(\Delta\omega t)} e^{-t\Gamma/\hbar}, \tag{S3}$$

with $\Delta\omega = \hbar\Delta E$ being the comb's frequency spacing caused by the Doppler shift. An exponential factor is added to account for the temporal response of a single comb tooth with spectral width Γ. The width of an absorber with optical thickness ζ is roughly given by $\Gamma = \Gamma_0(1+\zeta/4)$ (*54*), thus about 8 Γ_0 in our case. The actual temporal response of a single comb tooth, i.e. of a stainless-steel foil, is shown in Fig. S1 which is modified by a dynamical beat node at 70 ns.

As shown in Fig. S3A, the intensity distribution defined by Eq. (S3) reproduces the main features of the experimental data very well. More specifically, the position and width of the echoes are matching nearly perfectly, as well as the number of side maxima as a function of the teeth number. This indicates the excellent precision of the frequency comb setup. If one of the velocity transducers moves with a 5%-off velocity, the frequency comb formation is severely disturbed, as shown by simulations depicted in Fig. S3B. With such an imperfect velocity setting, the distinct side maxima structure between the echoes as visible in the measurements would not be noticeable anymore.

Estimation of losses in the storage process

The photons propagating through the frequency comb experience not only coherent resonant forward scattering, but also Compton scattering, photoelectric absorption, and incoherent resonant processes, such as inelastic phonon excitations, internal conversion, and nuclear fluorescence. Photons involved in these incoherent processes are lost for the storage process and do not appear in the measured histogram which counts only photons scattered in the forward direction. Hence, the incoherent processes are not included in the normalization factor N_{FS} used for the echo efficiency η_{echo} calculation. To estimate the incoherent losses of a nuclear system (e.g. the frequency comb), a direct relation between the incoming and outgoing photon pulse energy must be established. This

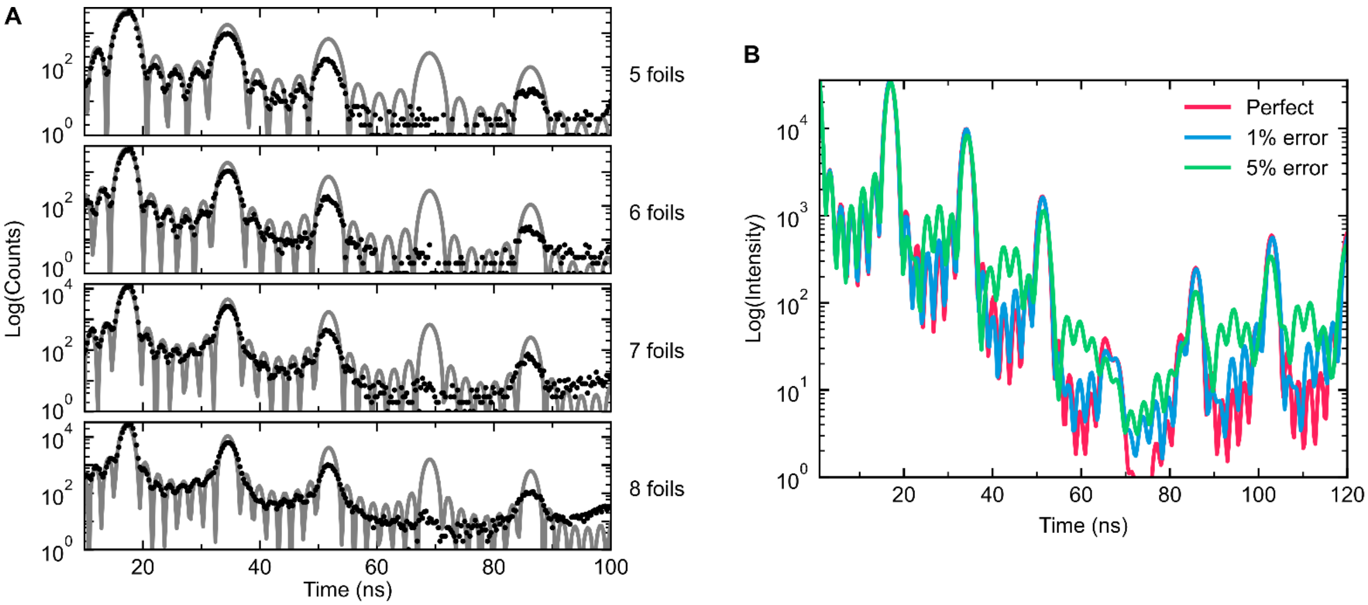

Fig. S3. Additional NFC characterization. (**A**) Measured decay histograms of frequency comb setups with varying number of foils, i.e. comb teeth, with a fixed velocity spacing of 5 mm/s (black). The decay patterns closely follow diffraction intensity patterns from a grating, see Eq. (S3) (gray, solid line). (**B**) Simulated decays of the frequency comb consisting of seven comb teeth with a fixed velocity spacing of 5 mm/s, wherein the drive supposed to run at 10 mm/s, is detuned by 0% ("perfect"), 1% or 5%.

can be done using the analytical response function of a thick nuclear resonant scatterer in the absence of hyperfine interactions (*56*),

$$R(t) = e^{-d\mu_e/2}\left(\delta(t) - \frac{\zeta}{2\tau_0} e^{-i(\omega_0-\omega_D)t} e^{-t/(2\tau_0)} \frac{J_1(\sqrt{x})}{\sqrt{x}}\,\theta(t)\right), \qquad \text{(S4)}$$

with μ_e being the electronic absorption coefficient due to Compton scattering and photoelectric absorption (for stainless-steel at the ^{57}Fe resonance energy: $\mu_e \simeq 0.048\ \mu\text{m}^{-1}$), $\delta(t)$ the Dirac delta distribution, τ_0 the natural lifetime of the excited state ($\tau_0 = 141$ ns), ω_D the motion-induced Doppler shift ($\omega_D = \Delta E_D/\hbar$) and $\theta(t)$ the Heaviside step function. The function, $J_1(\sqrt{x})/\sqrt{x}$, with its argument $x = \zeta t/\tau_0$, contains the Bessel function of first kind, J_1, which leads to an aperiodic dynamical beating for a medium with a large enough optical thickness. The optical thickness ζ carries information about the strength of incoherent-inelastic scattering via the Lamb-Mössbauer factor and of the internal conversion process which is included in the nuclear resonant cross section [Eq. (S3)].

With a photon with complex wave packet amplitude $\mathcal{E}_{\text{in}}$ impinging, and the foil's response function given, the complex photon wave packet emitted from the foil into the coherent resonant forward scattering channel is obtained by (*57*)

$$\mathcal{E}_{\text{out}}(t) = (R * \mathcal{E}_{\text{in}})(t), \qquad \text{(S5)}$$

where the operator "$*$" denotes a convolution. Eq. (S5) can be applied iteratively to calculate the propagation through multiple foils, namely through the seven Doppler detuned foils of the frequency comb. As a measure of the field energy, the time integrated intensity, $W = \int_0^\infty |\mathcal{E}(t)|^2 dt$, can be used to determine the total losses β_{tot} [defined in Eq. (4)] via,

$$\beta_{\text{tot}} = 1 - \frac{W_{\text{out}}}{W_{\text{in}}}. \qquad \text{(S6)}$$

In our case, the emitted wave packet from the superradiant cavity state, $\mathcal{E}_{\text{cav}}$, is selected as incoming photon wave packet, $\mathcal{E}_{\text{in}}$, since it is desired to be stored in the frequency comb. Note that the

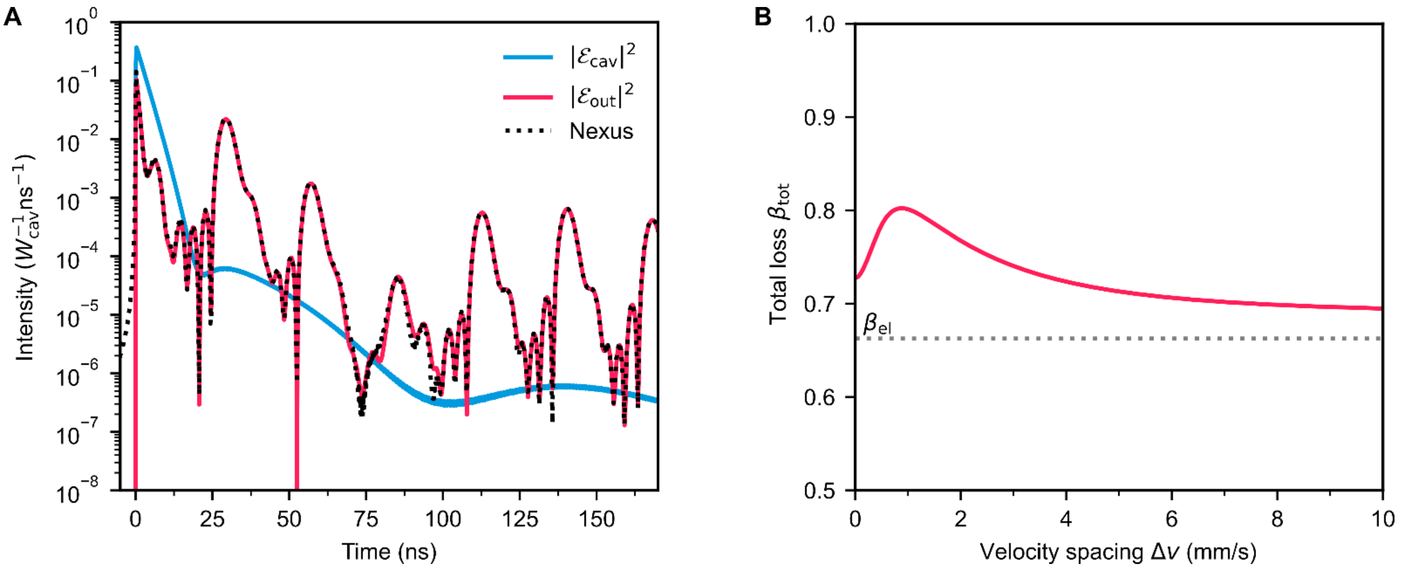

Fig. S4. Estimation of total losses. (**A**) Intensities of the photon wave packet emitted from the cavity decay, $\mathcal{E}_{cav}$, and from the combined setup (cavity and comb), $\mathcal{E}_{out}$, calculated via the analytical response function. The simulation of the emitted wave packet using *Nexus* is shown as a comparison. (**B**) Total energy loss after passing through the frequency comb calculated via the ratio of the time integrated outgoing to incoming intensity as a function of the velocity spacing. A lower limit is caused by the non-resonant electronic interaction, $\beta_{el} = 0.66$.

sequence of scatterers is irrelevant, as discussed in Methods in the main text. From the experiment, only the number of emitted photons by the cavity decay, $n_{cav}(t) \propto |\mathcal{E}_{cav}|^2$, are accessible (Figs. 3B and S2C). However, with the layer structure and hyperfine parameters determined via the fit routines, the complex amplitudes $\mathcal{E}_{cav}$ can be calculated by *Nexus*. In this calculation, the non-resonant interaction of the cavity with the synchrotron pulse is neglected, since only the resonant part is of interest for the storage procedure.

With $\mathcal{E}_{cav}$ determined from the simulation, the full propagation of $\mathcal{E}_{cav}$ through the frequency comb is calculated using Eq. (S5). The result is shown in Fig. S4A for a velocity spacing of 3.1 mm/s. The overall intensity scaling depends on an arbitrary scaling of $\mathcal{E}_{cav}$, which is here chosen to be $1/\sqrt{W_{cav}}$, so that the incoming energy is normalized to one. The relative scaling between $\mathcal{E}_{out}$ and $\mathcal{E}_{cav}$ is dictated by the analytical response function [Eq. (S4)], and therefore, is deterministic. The emitted photon wave packet can also be calculated using the numerical energy dependent scattering amplitudes obtained by *Nexus*. With the non-resonant contribution removed from the cavity scattering amplitude, the Fourier transformation of the product of all scattering amplitudes along the beam path (cavity and foils) results in a nearly identical outcome as obtained via the analytical response function, as shown in Fig. S4A.

The total losses β_{tot} as a function of the frequency comb's velocity spacing is shown in Fig. S4B. The overlap of the Doppler detuned absorption lines of the frequency comb depends on the velocity spacing. It is therefore not surprising that the coherent forward scattering intensity, and thus also its losses, strongly varies in the region where the lines strongly overlap (in between 0 mm/s and 1.5 mm/s). For higher velocities, the losses saturate, with a lower limit imposed by the non-resonant part, β_{el}, which is 66% for the given foil thicknesses at the ^{57}Fe resonance energy.

The calculated total losses were used to obtain the quantum memory efficiency depicted in Fig. 4A in the main text. For the derived optimal quantum memory performance at 3.1 mm/s, the total losses amount to $\beta_{tot} = 74\%$.

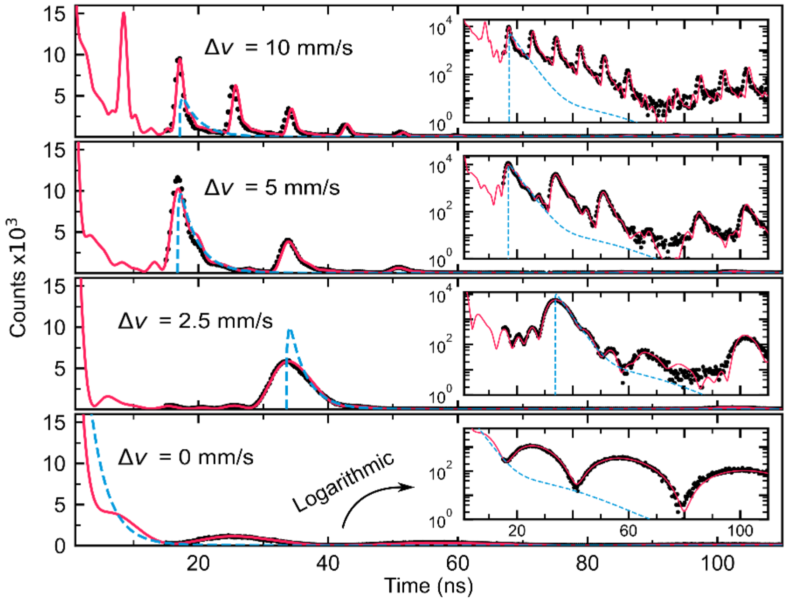

Fig. S5. Additional decay histograms for cavity-NFC setup. Additional decay histograms for four selected velocity spacings from Fig. 3A in linear and logarithmic scales. The patterns are simulated using the software package *Nexus* (red). The normalized cavity decay is overlaid (blue) at the first echo (second echo for $\Delta v = 10$ mm/s) with the normalization constant taken from the fidelity calculation, see Eq. (6).

Additional temporal responses from the cavity-frequency comb setup

Additional decay histograms for the cavity-frequency comb setup are depicted in Fig. S5. In all cases, except for $\Delta v = 0$ mm/s, the echo shapes reflect the shape of the photon wave packet emitted from the cavity, as discussed in the main text. For zero velocity, however, the time response of the cavity is only present at very early times due to the fast cavity decay. At late times, the time response corresponds to a 22 μm thick stainless-steel foil, as already seen in the response of the frequency comb setup (see Fig. 2B).

Comparison of histograms with and without cavity

A direct comparison between the results for the setup with and without cavity is shown in Fig. S6.

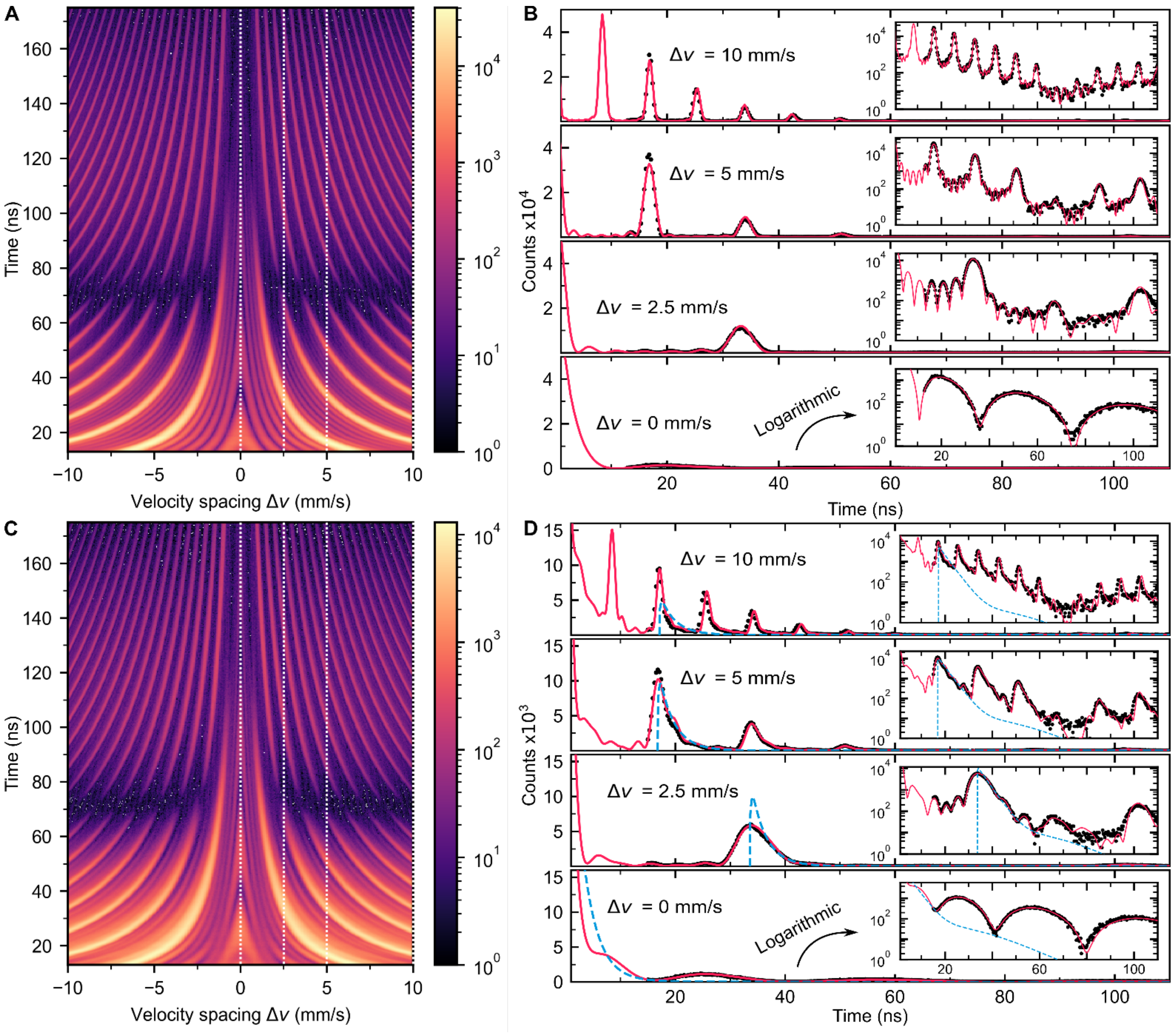

Fig. S6. Comparison of NFC response with and without cavity. Direct comparison of the two-dimensional histograms without (A) and with (C) cavity and the corresponding decay histograms at selected velocity spacings (B and D).

REFERENCES AND NOTES

1. A. I. Lvovsky, B. C. Sanders, W. Tittel, Optical quantum memory. *Nat. Photon* **3**, 706–714 (2009).

2. T. Chanelière, D. N. Matsukevich, S. D. Jenkins, S.-Y. Lan, T. A. B. Kennedy, A. Kuzmich, Storage and retrieval of single photons transmitted between remote quantum memories. *Nature* **438**, 833–836 (2005).

3. J. L. O'Brien, A. Furusawa, J. Vučković, Photonic quantum technologies. *Nat. Photon* **3**, 687–695 (2009).

4. M. K. Bhaskar, R. Riedinger, B. Machielse, D. S. Levonian, C. T. Nguyen, E. N. Knall, H. Park, D. Englund, M. Lončar, D. D. Sukachev, M. D. Lukin, Experimental demonstration of memory-enhanced quantum communication. *Nature* **580**, 60–64 (2020).

5. Y. Lei, F. K. Asadi, T. Zhong, A. Kuzmich, C. Simon, M. Hosseini, Quantum optical memory for entanglement distribution. *Optica* **10**, 1511–1528 (2023).

6. T. Chanelière, G. Hétet, N. Sangouard, "Chapter Two - Quantum optical memory protocols in atomic ensembles" in *Advances in Atomic, Molecular, and Optical Physics*, E. Arimondo, L. F. DiMauro, S. F. Yelin, Eds. (Academic Press, 2018; https://sciencedirect.com/science/article/pii/S1049250X18300028) vol. 67, pp. 77–150.

7. M. D. Lukin, Colloquium: Trapping and manipulating photon states in atomic ensembles. *Rev. Mod. Phys.* **75**, 457–472 (2003).

8. I. Novikova, R. Walsworth, Y. Xiao, Electromagnetically induced transparency-based slow and stored light in warm atoms. *Laser Photonics Rev.* **6**, 333–353 (2012).

9. D. Serrano, S. K. Kuppusamy, B. Heinrich, O. Fuhr, D. Hunger, M. Ruben, P. Goldner, Ultra-narrow optical linewidths in rare-earth molecular crystals. *Nature* **603**, 241–246 (2022).

10. A. Q. R. Baron, R. Rüffer, J. Metge, A fast, convenient, x-ray detector. *Nucl. Instrum. Methods Phys. Res., Sect. A* **400**, 124–132 (1997).

11. W.-T. Liao, A. Pálffy, C. H. Keitel, Coherent storage and phase modulation of single hard-x-ray photons using nuclear excitons. *Phys. Rev. Lett.* **109**, 197403 (2012).

12. X. Zhang, W.-T. Liao, A. Kalachev, R. Shakhmuratov, M. Scully, O. Kocharovskaya, Nuclear quantum memory and time sequencing of a single γ photon. *Phys. Rev. Lett.* **123**, 250504 (2019).

13. R. Röhlsberger, J. Evers, S. Shwartz, "Quantum and nonlinear optics with hard x-rays" in *Synchrotron Light Sources and Free-Electron Lasers: Accelerator Physics, Instrumentation and Science Applications*, E. Jaeschke, S. Khan, J. R. Schneider, J. B. Hastings, Eds. (Springer International Publishing, 2014; https://doi.org/10.1007/978-3-319-04507-8_32-1), pp. 1–28.

14. L. Bocklage, J. Gollwitzer, C. Strohm, C. F. Adolff, K. Schlage, I. Sergeev, O. Leupold, H.-C. Wille, G. Meier, R. Röhlsberger, Coherent control of collective nuclear quantum states via transient magnons. *Sci. Adv.* **7**, eabc3991 (2021).

15. Yu. V. Shvyd'ko, T. Hertrich, J. Metge, O. Leupold, E. Gerdau, H. D. Rüter, Reversed time in Mössbauer time spectra. *Phys. Rev. B* **52**, R711–R714 (1995).

16. Yu. V. Shvyd'ko, T. Hertrich, U. van Bürck, E. Gerdau, O. Leupold, J. Metge, H. D. Rüter, S. Schwendy, G. V. Smirnov, W. Potzel, P. Schindelmann, Storage of nuclear excitation energy through magnetic switching. *Phys. Rev. Lett.* **77**, 3232–3235 (1996).

17. X. Kong, A. Pálffy, Stopping narrow-band x-ray pulses in nuclear media. *Phys. Rev. Lett.* **116**, 197402 (2016).

18. P. Helistö, I. Tittonen, M. Lippmaa, T. Katila, Gamma echo. *Phys. Rev. Lett.* **66**, 2037–2040 (1991).

19. K. P. Heeg, A. Kaldun, C. Strohm, P. Reiser, C. Ott, R. Subramanian, D. Lentrodt, J. Haber, H.-C. Wille, S. Goerttler, R. Rüffer, C. H. Keitel, R. Röhlsberger, T. Pfeifer, J. Evers, Spectral narrowing of x-ray pulses for precision spectroscopy with nuclear resonances. *Science* **357**, 375–378 (2017).

20. K. P. Heeg, A. Kaldun, C. Strohm, C. Ott, R. Subramanian, D. Lentrodt, J. Haber, H.-C. Wille, S. Goerttler, R. Rüffer, C. H. Keitel, R. Röhlsberger, T. Pfeifer, J. Evers, Coherent x-ray−optical control of nuclear excitons. *Nature* **590**, 401–404 (2021).

21. R. N. Shakhmuratov, F. Vagizov, O. Kocharovskaya, Single gamma-photon revival from sandwich absorbers. *Phys. Rev. A* **87**, 013807 (2013).

22. F. Vagizov, V. Antonov, Y. V. Radeonychev, R. N. Shakhmuratov, O. Kocharovskaya, Coherent control of the waveforms of recoilless γ-ray photons. *Nature* **508**, 80–83 (2014).

23. Y. V. Radeonychev, I. R. Khairulin, F. G. Vagizov, M. Scully, O. Kocharovskaya, Observation of acoustically induced transparency for γ-ray photons. *Phys. Rev. Lett.* **124**, 163602 (2020).

24. R. N. Shakhmuratov, F. G. Vagizov, V. A. Antonov, Y. V. Radeonychev, M. O. Scully, O. Kocharovskaya, Transformation of a single-photon field into bunches of pulses. *Phys. Rev. A* **92**, 023836 (2015).

25. I. R. Khairulin, Y. V. Radeonychev, O. Kocharovskaya, Slowing down x-ray photons in a vibrating recoilless resonant absorber. *Sci. Rep.* **12**, 20270 (2022).

26. I. R. Khairulin, Y. V. Radeonychev, O. Kocharovskaya, Compression of the synchrotron Mössbauer x-ray photon waveform in an oscillating resonant absorber. *Photonics* **9**, 829 (2022).

27. X. Zhang, A. A. Svidzinsky, Superradiant control of γ-ray propagation by vibrating nuclear arrays. *Phys. Rev. A* **88**, 033854 (2013).

28. R. Röhlsberger, H.-C. Wille, K. Schlage, B. Sahoo, Electromagnetically induced transparency with resonant nuclei in a cavity. *Nature* **482**, 199–203 (2012).

29. R. Röhlsberger, K. Schlage, B. Sahoo, S. Couet, R. Rüffer, Collective lamb shift in single-photon superradiance. *Science* **328**, 1248–1251 (2010).

30. J. Haber, X. Kong, C. Strohm, S. Willing, J. Gollwitzer, L. Bocklage, R. Rüffer, A. Pálffy, R. Röhlsberger, Rabi oscillations of x-ray radiation between two nuclear ensembles. *Nat. Photon* **11**, 720–725 (2017).

31. J. Haber, K. S. Schulze, K. Schlage, R. Loetzsch, L. Bocklage, T. Gurieva, H. Bernhardt, H.-C. Wille, R. Rüffer, I. Uschmann, G. G. Paulus, R. Röhlsberger, Collective strong coupling of x-rays and nuclei in a nuclear optical lattice. *Nat. Photon* **10**, 445–449 (2016).

32. K. P. Heeg, H.-C. Wille, K. Schlage, T. Guryeva, D. Schumacher, I. Uschmann, K. S. Schulze, B. Marx, T. Kämpfer, G. G. Paulus, R. Röhlsberger, J. Evers, Vacuum-assisted generation and control of atomic coherences at x-ray energies. *Phys. Rev. Lett.* **111**, 073601 (2013).

33. M. Afzelius, C. Simon, H. de Riedmatten, N. Gisin, Multimode quantum memory based on atomic frequency combs. *Phys. Rev. A* **79**, 052329 (2009).

34. D. Lago-Rivera, S. Grandi, J. V. Rakonjac, A. Seri, H. de Riedmatten, Telecom-heralded entanglement between multimode solid-state quantum memories. *Nature* **594**, 37–40 (2021).

35. H. de Riedmatten, M. Afzelius, M. U. Staudt, C. Simon, N. Gisin, A solid-state light–matter interface at the single-photon level. *Nature* **456**, 773–777 (2008).

36. H.-C. Wille, H. Franz, R. Röhlsberger, W. A. Caliebe, F.-U. Dill, Nuclear resonant scattering at PETRA III: Brillant opportunities for nano – and extreme condition science. *J. Phys. Conf. Ser.* **217**, 012008 (2010).

37. R. Rüffer, A. I. Chumakov, Nuclear resonance beamline at ESRF. *Hyperfine Interact.* **97-98**, 589–604 (1996).

38. Yu. Kagan, Theory of coherent phenomena and fundamentals in nuclear resonant scattering. *Hyperfine Interact.* **123**, 83–126 (1999).

39. J. P. Hannon, G. T. Trammell, Coherent γ-ray optics. *Hyperfine Interact.* **123**, 127–274 (1999).

40. G. V. Smirnov, General properties of nuclear resonant scattering. *Hyperfine Interact.* **123**, 31–77 (1999).

41. L. Bocklage, Nexus - Nuclear Elastic X-ray Scattering Universal Software, Zenodo (2023); https://doi.org/10.5281/zenodo.7716207.

42. W. Sturhahn, E. Gerdau, Evaluation of time-differential measurements of nuclear-resonance scattering of x rays. *Phys. Rev. B* **49**, 9285–9294 (1994).

43. X. Zhang, Exact solution of gradient echo memory and analytical treatment of gradient frequency comb. arXiv:1602.05115 [quant-ph] (2016). https://doi.org/10.48550/arXiv.1602.05115.

44. M. F. Askarani, A. Das, J. H. Davidson, G. C. Amaral, N. Sinclair, J. A. Slater, S. Marzban, C. W. Thiel, R. L. Cone, D. Oblak, W. Tittel, Long-lived solid-state optical memory for high-rate quantum repeaters. *Phys. Rev. Lett.* **127**, 220502 (2021).

45. P. Jobez, N. Timoney, C. Laplane, J. Etesse, A. Ferrier, P. Goldner, N. Gisin, M. Afzelius, Towards highly multimode optical quantum memory for quantum repeaters. *Phys. Rev. A* **93**, 032327 (2016).

46. C. Clausen, I. Usmani, F. Bussières, N. Sangouard, M. Afzelius, H. de Riedmatten, N. Gisin, Quantum storage of photonic entanglement in a crystal. *Nature* **469**, 508–511 (2011).

47. Z.-Q. Zhou, J. Wang, C.-F. Li, G.-C. Guo, Efficient spectral hole-burning and atomic frequency comb storage in Nd3+:YLiF4. *Sci. Rep.* **3**, 2754 (2013).

48. Y. Shvyd'ko, R. Röhlsberger, O. Kocharovskaya, J. Evers, G. A. Geloni, P. Liu, D. Shu, A. Miceli, B. Stone, W. Hippler, B. Marx-Glowna, I. Uschmann, R. Loetzsch, O. Leupold, H.-C. Wille, I. Sergeev, M. Gerharz, X. Zhang, C. Grech, M. Guetg, V. Kocharyan, N. Kujala, S. Liu, W. Qin, A. Zozulya, J. Hallmann, U. Boesenberg, W. Jo, J. Möller, A. Rodriguez-Fernandez, M. Youssef, A. Madsen, T. Kolodziej, Resonant x-ray excitation of the nuclear clock isomer Sc. *Nature* **622**, 1–5 (2023).

49. S. Kraemer, J. Moens, M. Athanasakis-Kaklamanakis, S. Bara, K. Beeks, P. Chhetri, K. Chrysalidis, A. Claessens, T. E. Cocolios, J. G. M. Correia, H. D. Witte, R. Ferrer, S. Geldhof, R. Heinke, N. Hosseini, M. Huyse, U. Köster, Y. Kudryavtsev, M. Laatiaoui, R. Lica, G. Magchiels, V. Manea, C. Merckling, L. M. C. Pereira, S. Raeder, T. Schumm, S. Sels, P. G. Thirolf, S. M. Tunhuma, P. Van Den Bergh, P. Van Duppen, A. Vantomme, M. Verlinde, R. Villarreal, U. Wahl, Observation of the radiative decay of the 229Th nuclear clock isomer. *Nature* ***617***, 706–710 (2023).

50. J. Tiedau, M. V. Okhapkin, K. Zhang, J. Thielking, G. Zitzer, E. Peik, F. Schaden, T. Pronebner, I. Morawetz, L. T. De Col, F. Schneider, A. Leitner, M. Pressler, G. A. Kazakov, K. Beeks, T. Sikorsky, T. Schumm, Laser excitation of the Th-229 nucleus. *Phys. Rev. Lett.* **132**, 182501 (2024).

51. P. Schindelmann, U. van Bürck, W. Potzel, G. V. Smirnov, S. L. Popov, E. Gerdau, Yu. V. Shvyd'ko, J. Jäschke, H. D. Rüter, A. I. Chumakov, R. Rüffer, Radiative decoupling and coupling of nuclear oscillators by stepwise Doppler-energy shifts. *Phys. Rev. A* **65**, 023804 (2002).

52. G. V. Smirnov, U. van Bürck, W. Potzel, P. Schindelmann, S. L. Popov, E. Gerdau, Yu. V. Shvyd'ko, H. D. Rüter, O. Leupold, Propagation of nuclear polaritons through a two-target system: Effect of inversion of targets. *Phys. Rev. A* **71**, 023804 (2005).

53. Y.-H. Chen, M.-J. Lee, I.-C. Wang, S. Du, Y.-F. Chen, Y.-C. Chen, I. A. Yu, Coherent optical memory with high storage efficiency and large fractional delay. *Phys. Rev. Lett.* **110**, 083601 (2013).

54. R. Röhlsberger, "Coherent elastic nuclear resonant scattering" in *Nuclear Condensed Matter Physics with Synchrotron Radiation* (Springer Tracts in Modern Physics, vol.208, Springer-Verlag Berlin Heidelberg, 2004), pp. 67–180.

55. B. Sahoo, K. Schlage, J. Major, U. von Hörsten, W. Keune, H. Wende, R. Röhlsberger, Preparation and characterization of ultrathin stainless steel films. *AIP Conf. Proc.* **1347**, 57–60 (2011).

56. G. V. Smirnov, V. G. Kohn, Theory of nuclear resonant scattering of synchrotron radiation in the presence of diffusive motion of nuclei. *Phys. Rev. B* **52**, 3356–3365 (1995).

57. B. Herkommer, J. Evers, Phase-sensitive nuclear target spectroscopy. *Phys. Rev. Res.* **2**, 023397 (2020).
